# Effect of a Photolithography Polymer Mask’s Dynamic Viscoelasticity on Microchannel Cross-Sectional Shapes of Glass Processed by Micropowder Blasting †

**DOI:** 10.3390/mi15020256

**Published:** 2024-02-09

**Authors:** Mikinari Takada, Mao Hamamoto, Hiromasa Yagyu

**Affiliations:** Department of Mechanical Engineering, Kanto Gakuin University, Yokohama 236-8501, Japan

**Keywords:** microchannels, photolithography, processing, erosion, viscoelasticity

## Abstract

In this study, a micropowder blasting system with varying processing temperatures was proposed to control the cross-sectional shape of a channel processed on a glass substrate. Based on an analysis of the processing temperature-dependence of the dynamic viscoelastic properties of a commercial mask material for micropowder blasting, a processing temperature control system that can be installed in a micropowder blasting machine was designed. The erosion of the mask during micropowder blasting depended on the loss tangent in dynamic viscoelasticity, and showed a maximum value at a processing temperature of 100 °C. Moreover, we confirmed that the maximum decrease in the width of the processed microchannel was 30 µm (12%) by mask erosion, and this change was large compared with the maximum change in the thickness of the eroded mask. These results clarified that varying the processing temperature using a mask could control the cross-section of the processed line pattern profile on glass, and a small-width channel was realized at a processing temperature of 109 °C.

## 1. Introduction

Several processing techniques, such as drilling [1], lasering [2], wet etching [3,4], dry etching [5,6,7], and micropowder blasting [8,9,10], have been used in glass micromachining. Drilling and laser methods are used to produce large patterns. Wet etching yields a smooth surface. However, a microstructure with a high aspect ratio cannot be obtained. Dry etching can also be used to obtain precise microstructures. However, the process requires a complex fabrication system. Electrochemical discharge machining (EDCM) is a hybrid machining process of electrodischarge machining (EDM) and electrochemical machining (ECM), in which the material is removed through thermal energy melting [11,12]. EDCM can be processed as both conducting and nonconducting, and a microchannel can be machined on a glass substrate. Spark-assisted chemical engraving (SACE) is a micromachining technology based on electrochemical discharge for the low-cost machining of microholes and microchannels in glass [13]. However, these machining processes require an electrolyte solution to process the workpiece, and the processed shape depends on the flow state of the electorate. Micropowder blasting can be used to process high-aspect-ratio microstructures; however, microstructures have rough surfaces, and the fabrication of small patterns below 100 μm is difficult with this technique.

Micropowder blasting is an abrasive jet machining technique that is used to process brittle substrates, such as glass and silicon wafers, and has been used in flat panel displays (FPD) [8,14], microelectromechanical systems (MEMS) [9], and micro total analysis systems (MicroTAS) [10,15,16]. Among them, micropowder blasting is applied to process microchannels on glass and silicon at a low cost, and can be realized in low-cost chemical reaction systems. Because the processing depth depends on the inclination angle of the nozzle [17,18,19], a three-dimensional channel pattern can be processed by varying the inclination of the nozzle [16]. However, this method requires a dedicated system to control the nozzle’s inclination angle.

Controlling the shape of a processed structure via micropowder blasting is crucial because different aspect ratios and cross-sectional shapes of the microchannel are required for different applications. During the processing of glass by micropowder blasting, a mask pattern was fabricated on the substrate, and microparticles were accelerated toward the sample with high-pressure airflow from the nozzle. The substrate (or nozzle) was scanned repeatedly to process the entire glass substrate surface. Photolithography is required to create fine mask patterns on a substrate, and uses photoresists produced from polymers that are less eroded by micropowder blasting [17,20]. In polymer dynamics, the amount of wear of polymer materials is inversely proportional to their impact resilience [21].

A polymer material used as a photolithography resist is a viscoelastic material that has elasticity and viscosity. In dynamic viscoelasticity, elasticity and viscosity are measured as storage modulus and loss modulus, respectively. In addition, loss tangent is calculated as a ratio of storage modulus to loss modulus. These properties depend on temperature, and at the peak of loss tangent, as a particle cannot rebound from the mask during micropowder blasting, the erosion of the mask is large. Erosion of the mask material by micropowder blasting depends on the dynamic viscoelastic properties of the mask, which affect the processing temperature [22]. This confirms that polyester polyurethane with a low peak temperature of loss tangent, lower than room temperature in viscoelastic properties, has high erosion resistance to micropowder blasting. However, this polymer material is a specialized mask that can be processed using a low-power laser and cannot be used to make micropatterns by photolithography, excluding reports by the authors of [23]. A typical mask material used in micropowder blasting is a thick photoresist comprising photosensitive polyurethane. These photoresists can be fabricated on substrates using photolithography. However, the dependence of dynamic viscoelastic properties on the processing shapes of these photoresists has not yet been clarified.

In this study, the processing temperature dependence of the erosion of a commercial micropowder blasting mask made of an elastic negative photoresist film was clarified for the first time using a temperature control system designed based on the measured dynamic viscoelastic properties of the mask. Moreover, we confirmed the effect of changing the processing temperature on the processed cross-sectional shape of the line pattern on the glass.

## 2. Materials and Methods

### 2.1. Materials

Soda-lime glass (S1225, Matsunami glass Ind. Ltd., Kishiwada, Japan, 25 × 75 × 1.2 mm) was used as the substrate. An elastic negative-resist film (MS7050, Mitsubishi Paper Mills Ltd., Tokyo, Japan) was used as the mask material. The thickness of the photoresist film was 50 μm.

**Figure 1:**
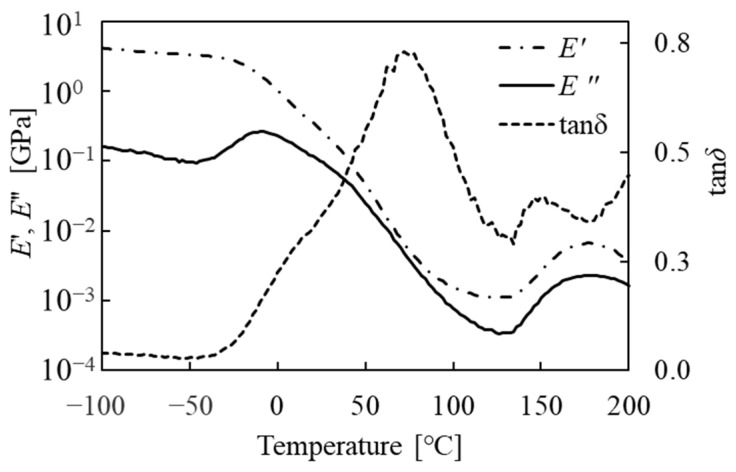
Dynamic viscoelastic properties (storage modulus *E*′, loss modulus *E*″, and loss tangent tanδ) as functions of temperature.

Figure 1 shows the dynamic viscoelastic properties, storage modulus (*E*′), loss modulus (*E*″), and loss tangent (tanδ) of the exposed photoresist film (30 mJ/cm^2^) with a width of 5 mm and a length of 25 mm. Measurement conditions were a temperature range from −100 to 200 °C, a dynamic strain of 0.03%, a gauge length of 15 mm, and a frequency of 10 Hz. Dynamic viscoelastic properties were measured using a dynamic mechanical analysis instrument (Rheogel-E4000, UBM, Kyoto, Japan). The peak temperatures of the loss modulus and loss tangent were −10 and 80 °C, respectively. The peak temperature of the loss modulus was approximately zero at 50 °C.

### 2.2. Mask Pattern Fabrication

**Figure 2:**
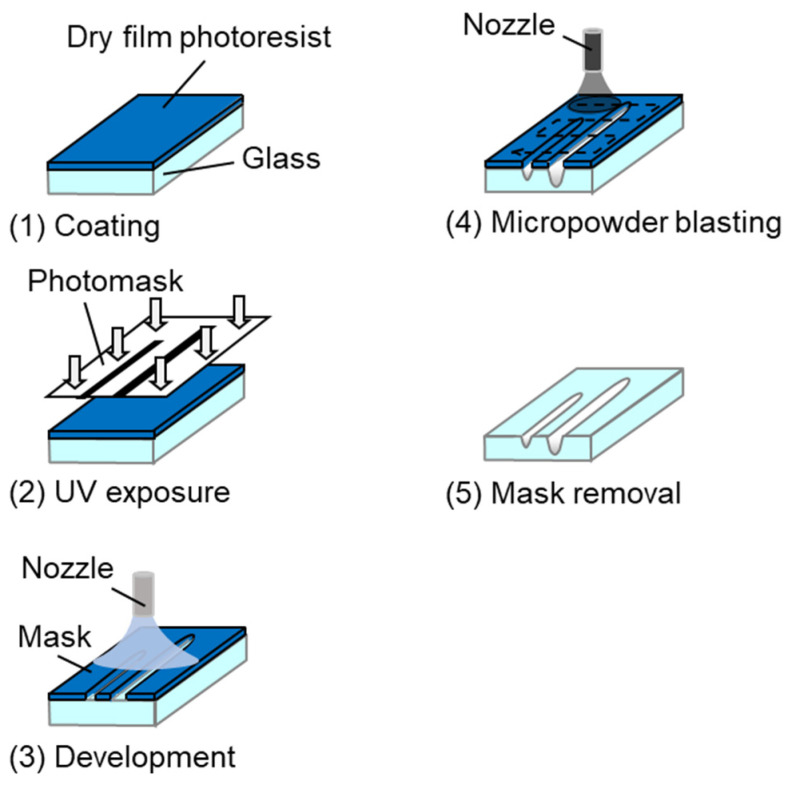
Schematic of the micropowder blasting process for the fabrication of microchannels on glass.

Figure 2 shows the micropowder blasting process used to fabricate microchannels on glass. The line pattern mask on the glass substrate was fabricated using photolithography as follows: the dry film resist was coated onto a glass substrate (Figure 2(1)) and exposed to ultraviolet irradiation through a line pattern photomask with L/S 200 μm (Figure 2(2)). The exposed substrate was developed by spraying a developer solution (0.2% NaCO_3_) before rinsing with distilled water and drying with N_2_ air to fabricate a mask pattern on the glass (Figure 2(3)). Subsequently, the substrate with the mask was processed by micropowder blasting using Al_2_O_3_ microparticles (Figure 2(4)). Finally, mask removal was performed by immersing the mask in a remover solution (3% NaOH) (Figure 2(5)), as required.

### 2.3. Micropowder Blasting

**Figure 3:**
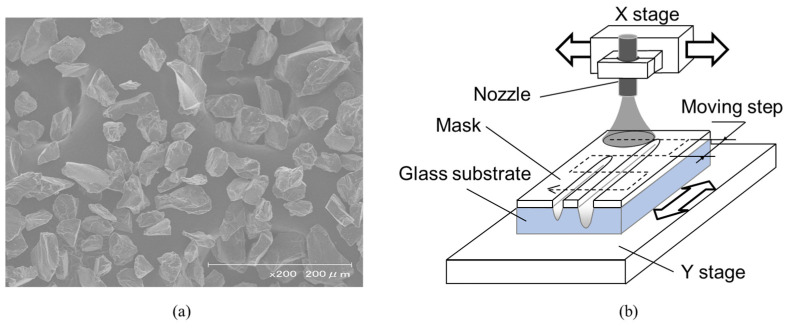
(**a**) SEM image of Al_2_O_3_ microparticles used for micropowder blasting. (**b**) Schematic of the micropowder blasting process.

The micropowder blasting system was developed using a fine sandblasting unit (Basic Mobil, Renfert GmbH, Hilzingen, Germany) for blasting the substrate and a 3D milling system (MDX-40A, Roland DG Corporation, Hamamatsu, Japan) with XYZ motorized stages of 0.002 mm resolution for scanning the nozzle. This system allowed control of the nozzle position and blasting time using the NC code by replacing the spindle unit of the 3D milling system with the nozzle of the sandblasting unit and attaching an electromagnetic air valve to the sandblasting unit. Table 1 lists the micropowder blasting conditions used in this study. Al_2_O_3_ microparticles with a mean diameter of 25 μm (Cobra white, Renfert GmbH, Hilzingen, Germany) were ejected from the nozzle (diameter, 0.8 mm) toward the sample with high-pressure airflow (air pressure, 0.30 MPa) at an incident angle of 90°. The microparticles had extremely sharp edges and a purity of 99.7% Al_2_O_3_, as shown in Figure 3a.

**Figure 4:**
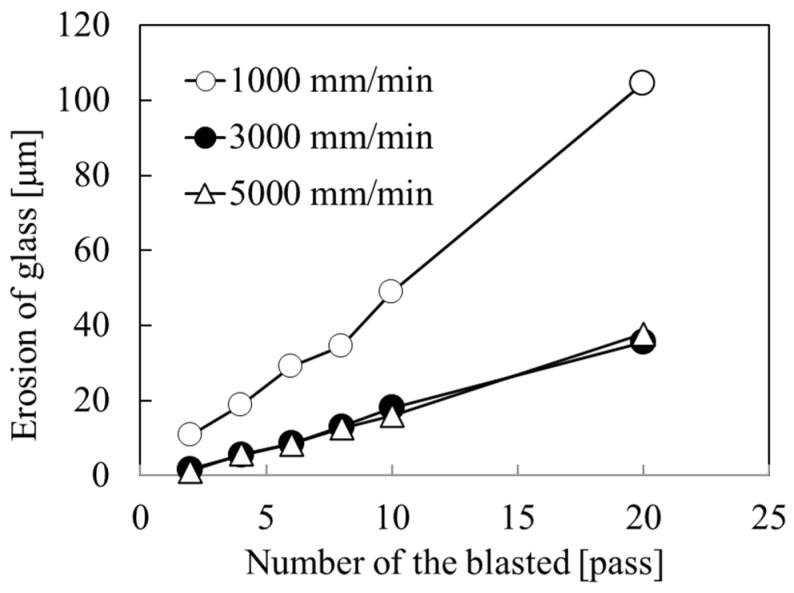
Erosion of glass as a function of the number of micropowder blasting passes for scan speeds of 1000, 3000, and 5000 mm/min.

To process the entire surface of the glass substrate, the nozzle was scanned repeatedly, as shown in Figure 3b. The scanning system used in this study comprised an X stage for moving the nozzle and a Y stage for moving the step. Hereafter, each scanning session is referred to as a “pass” by the X stage with a constant velocity and a moving step. The scan speed was set to 1000 mm/min because the erosion of the glass increased linearly with the number of blasting passes, and large erosion was obtained at a scan speed of 1000 mm/min (Figure 4). The erosion distribution of glass, as shown in Figure 4, was small, and the standard deviation ranged from 0.6 to 1.1 mm for 1000 mm/min, from 0.2 to 0.5 mm for 3000 mm/min, and from 0.1 to 0.6 mm for 5000 mm/min. In addition, the long processing time required for the sample that was 26 mm in width and 75 mm in length increased, and a single micropowder blasting pass took approximately two minutes when the scanning area was set to 50 mm in width and 100 mm in length.

The blasting area (diameter) of the sample was 10 mm at a distance of 90 mm from the nozzle to the sample surface. The surface of the processed glass substrate was uneven when the step size was 10 mm, and a uniform processing surface was obtained when the step size was 5 mm. Therefore, the moving step was set to 5 mm in this study. When the glass substrate coated with the patterned polymer mask was hit by the Al_2_O_3_ microparticles, it was machined in the open areas of the polymer mask owing to the difference in the erosion rate between the polymer mask and the exposed glass substrate. To perform micropowder blasting using one substrate at 10, 20, and 30 micropowder blasting passes, the fabricated substrate was masked using four polyvinyl tapes. After 10 passes of micropowder blasting, the polyvinyl mask was removed from the substrate and the same substrate was processed for the next 10 passes. Subsequently, the processed glass was immersed in an ultrasonic bath to remove excess particles. After cleaning, we evaluated the processed glass because microparticles and mask debris were not observed in the channel.

### 2.4. Temperature Control System

**Figure 5:**
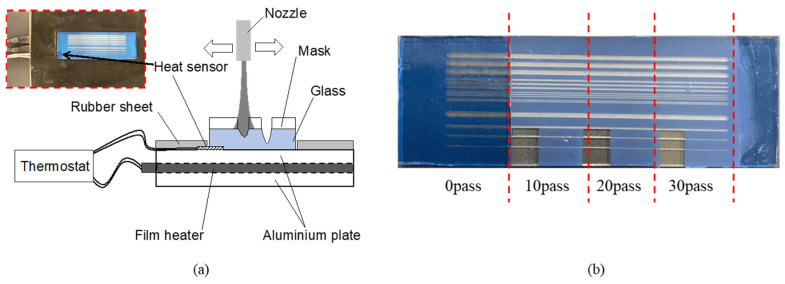
(**a**) Schematic and photograph of the temperature control system of micropowder blasting. The photograph shows the setup section of the glass substrate. (**b**) Substrate with the mask processed via micropowder blasting using 0, 10, 20, and 30 passes.

From the peak temperature of the loss tangent in Figure 1, and because the maximum erosion of the photoresist film was expected at a temperature of 80 °C, a temperature control system was produced using a film heater (PIFH-TC, Ichinen Manufacturing Co., Ltd., Nagoya, Japan, 200 mm × 100 mm) with a maximum operating temperature of 200 °C. Figure 5a shows the temperature control system, which comprised a film heater, aluminum plates with a thickness of 1 mm, a heat sensor (TH-8297-1, ThreeHigh, Yokohama, Japan), and a thermostat. The film heater was sandwiched between two aluminum plates, and the surface of the plate was covered with a rubber seat. To set up the substrate on an aluminum plate, a rubber seat was cut into a fixed area of the glass substrate. The film heater was controlled such that the measured processing temperature was set to the temperature of the thermostat. A temperature control system was installed in the micropowder blasting machine, and glass with a mask pattern was processed.

### 2.5. Evaluation Method

The substrate with the mask was divided into three areas, and then masked with vinyl tape (which had a high micropowder blasting resistance); the vinyl tape was peeled off after every 10 blasting passes to process 10, 20, and 30 passes, as shown in Figure 5b. The erosion of the processed mask was measured as the step height between the unprocessed glass surface removed from the mask and the processed section of the mask, using a surface roughness tester (Surfcom480A, Tokyo Seimitsu Co., Ltd., Hachioji, Japan). The cross-section of the line pattern processed on the glass substrate was observed using a confocal laser microscope (VK-8500; Keyence Corporation, Osaka, Japan).

## 3. Results and Discussion

### 3.1. Mask Erosion

Table 2 lists the set temperatures on the thermostat and the processing temperatures measured by the heat sensor. The processing temperature decreased due to micropowder blasting, and the processing temperature was 109 °C at the set temperature of 130 °C owing to airflow from the nozzle during processing.

**Figure 6:**
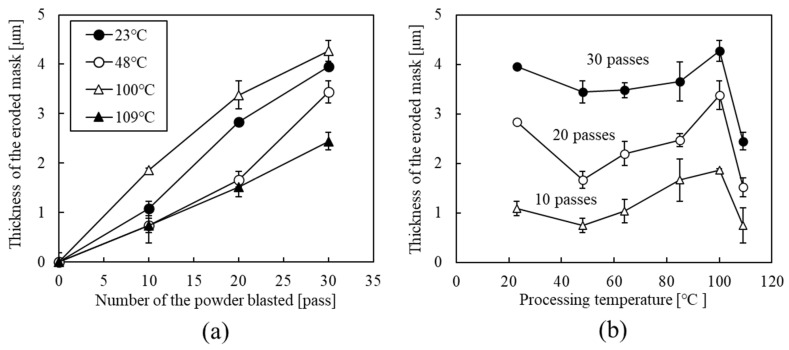
(**a**) Erosion of the mask’s thickness as a function of the number of micropowder blasting passes using Al_2_O_3_ microparticles at an air pressure of 0.30 MPa. (**b**) Erosion of the mask’s thickness as a function of the processing temperature for 10, 20, and 30 micropowder blasting passes.

Figure 6a shows the erosion of the mask thickness as a function of the number of micropowder blasting passes. The erosion of the mask thickness depended on the processing temperature and proportionally increased with the number of micropowder blasting passes.

Figure 6b shows the erosion of the mask thickness as a function of processing temperature. The erosion of the mask thickness at 10, 20, and 30 micropowder blasting passes exhibited maximum values at the processing temperature of 100 °C and minimum values at the processing temperature of 109 °C. The erosion of the mask thickness decreased at a processing temperature of 48 °C, but subsequently increased at a processing temperature of 23 °C. These erosion tendencies were in good agreement with the viscoelastic properties, including *E*″ and tan*δ*. We assumed that the difference in the maximum temperature of the loss tangent and the processing temperature at the maximum value of processing was attributed not to sensing the processing temperature, but to sensing the substrate temperature by the heat sensor. Therefore, the erosion of the mask depended on *E*″ and tan*δ* (Figure 1 and Figure 6b), and changing the processing temperature was expected to control the channel profile in the glass substrate.

### 3.2. Line Pattern Profile

**Figure 7:**
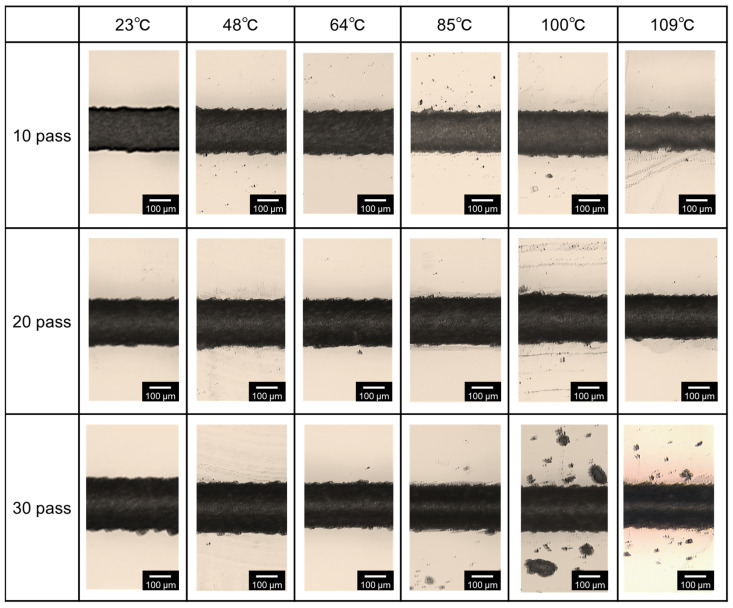
Top-view photographs of processed line patterns on glass at 10, 20, and 30 micropowder blasting passes and processing temperatures of 23, 48, 64, 80, 100, and 109 °C.

Figure 7 shows top-view photographs of the line patterns processed on the glass after micropowder blasting for 10, 20, and 30 passes. For micropowder blasting with 10 passes, the line pattern width depended on the processing temperature, and a large line width appeared at a processing temperature of 100 °C. These results confirmed that mask erosion affected the processing width of the glass. In contrast, in cases with micropowder blasting for 20 and 30 passes, no large changes in the line pattern width appeared.

**Figure 8:**
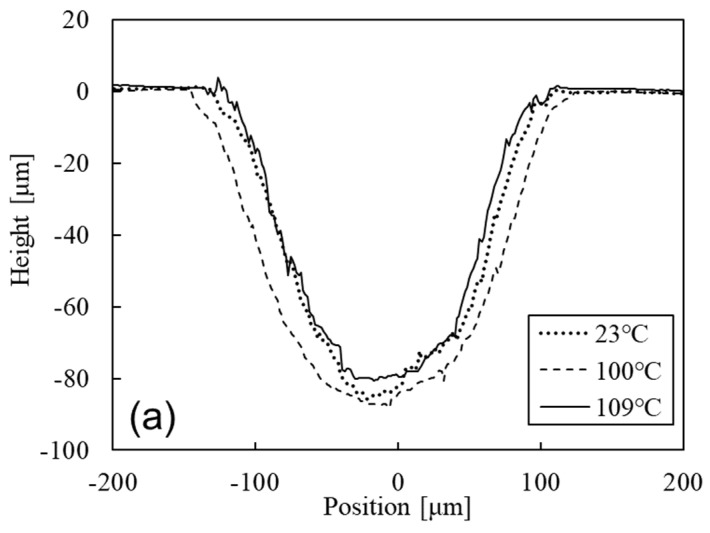

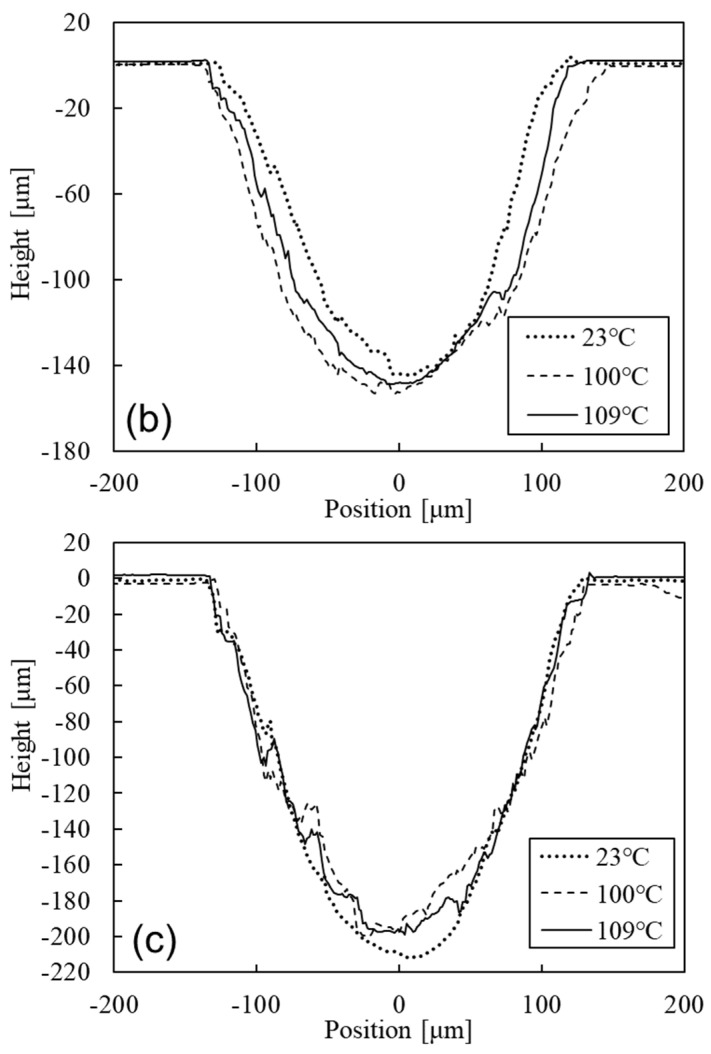
Cross-sectional shapes of fabricated channels on glass using a 200 μm width line pattern mask for micropowder blasting with (**a**) 10, (**b**) 20, and (**c**) 30 passes.

Figure 8 shows the cross-sectional shapes of the processed channels on the glass at processing temperatures of 23, 100, and 109 °C that were micropowder blasted with 10, 20, and 30 passes, respectively. We confirmed that the roughness of the channel tended to change when the number of micropowder blasting passes increased. We assumed that the roughness of the channel of glass was large because the reflection of the blasted particle occurred as the number of micropowder blasting passes increased. Results from 10 passes of micropowder blasting (Figure 8a) confirmed that the cross-sectional profile was controlled by varying the processing temperature during micropowder blasting, because the erosion of the mask in the lateral direction against the mask’s open width decreased. However, results from micropowder blasting with 20 and 30 passes (Figure 8b,c) confirmed that changes in the cross-sectional profile were small in comparison with those of 10 passes, and the processed width at 30 passes was not changed by the processing temperature. We assumed that this was attributed to decreasing the stiffness of the mask material by the micropowder blasting process, as many abrasion scratches were observed on a masked area of the glass at the micropowder blasting of 30 passes, which was changed by processing temperatures of 100 and 109 °C, as shown in Figure 7.

**Figure 9:**
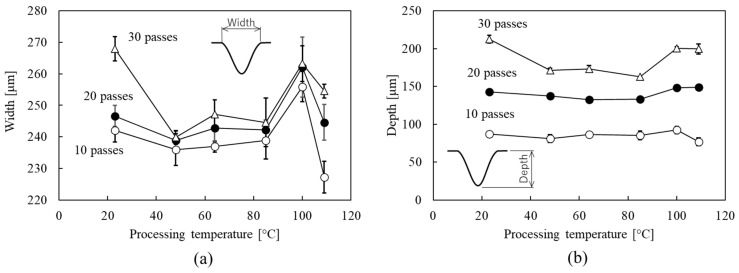
(**a**) Widths of processed channels and (**b**) depths of processed channels using a 200 μm width line pattern mask during micropowder blasting with 10, 20, and 30 passes as a function of the processing temperature.

Figure 9 shows the width and depth of the processed channel using a 200 μm width line pattern mask for micropowder blasting with 10, 20, and 30 passes as a function of the processing temperature. The result of micropowder blasting with 10 passes confirmed that the processed channel widths on the glass were 242 ± 3.7, 233 ± 5.0, 256 ± 4.7, and 227 ± 5.0 μm at processing temperatures of 23, 48, 100, and 109 °C, respectively. The smallest width of a processed channel was realized at the processing temperature of 109 °C. The maximum change in the processed width was 30 µm (12%), and this change was indicated by a large value in comparison with maximum changes in the thickness of the eroded mask (1.1 μm), as shown in Figure 9. Results of micropowder blasting with 20 and 30 passes confirmed that the processed channel widths on the glass showed a minimum value at the processing temperature of 48 °C.

The largest depth of a processed channel at micropowder blasting with 20 passes was 148 ± 3.9 μm at the processing temperature of 100 °C. In the case of micropowder blasting with 30 passes, the depth at the processing temperature of 23 °C showed a maximum value. These results indicate that processing at more than 10 micropowder blasting passes could not realize an effective cross-sectional change of the processed channel by controlling the processing temperature.

However, we found that the cross-sectional shape of the processed channel could be effectively controlled by varying the processing temperature of micropowder blasting with 10 passes. Nielson reported that the erosion of a polymer by micropowder blasting depended on the incident angle of the microparticles, and that this erosion showed a maximum value at an incident angle of 25° [24]. Therefore, the high erosion of the sidewall compared to that of the mask thickness in Figure 9 was attributed to the inclination of the sidewall in the mask-opening area. This phenomenon has been elucidated by simulating micropowder blasting [25,26].

Because the loss modulus decreased at a processing temperature of 48 °C, as shown in Figure 1, we assumed that the processing temperature variation of the loss modulus would affect the erosion of processed mask material. In addition, as explained in the Introduction, we expected particle rebound to reach its minimum at the peak of loss tangent. Therefore, the erosion of the mask material decreased at the processing temperature of approximately 48 °C owing to the decrease in the loss modulus; it then increased to approximately 109 °C owing to the increase in the peak temperature of the loss tangent. From the relationship between the processing temperature and erosion of the mask, we concluded that the opening width of the mask pattern was increased by the micropowder blasting process, and the processing width on the glass substrate was changed.

These results revealed that the cross-sectional shape of the glass could be controlled by varying the processing temperature of the commercial elastic negative photoresist film during micropowder blasting.

## 4. Conclusions

A temperature control system that could be installed in a micropowder blasting machine was developed by considering the viscoelastic properties of the mask material, and the processing temperature dependence of the erosion of the mask thickness was evaluated. Experimental results confirmed that the erosion of the mask thickness was at a maximum at a processing temperature of 100 °C, and at a minimum at a processing temperature of 109 °C. Evaluations of processed line pattern widths confirmed that the maximum changes in the processed width were 30 µm (12%), and micropowder blasting with temperature control was attributed to the realization of a channel with a smaller width compared with the channel processed at room temperature.

## Figures and Tables

**Table 1:** Conditions of micropowder blasting.

Material of the Microparticle	Al_2_O_3_
Microparticle mean diameter	25 μm
Blasted pressure	0.30 MPa
Distance from the sample	90 mm
Nozzle diameter	0.8 mm
Stage moving speed	1000 mm/min
Stage moving step	5 mm
Amount of blasted	2 g/min

**Table 2:** Set temperatures and processing temperatures.

Set Temperatures [°C]	Processing Temperatures [°C]
23	23
50	48
70	64
90	85
110	100
130	109

## Data Availability

Original raw data and datasets generated during this study are available from the corresponding author upon reasonable request for noncommercial purposes.
